# Covalent Defects Restrict Supramolecular Self-Assembly of Homopolypeptides: Case Study of β_2_-Fibrils of Poly-L-Glutamic Acid

**DOI:** 10.1371/journal.pone.0105660

**Published:** 2014-08-21

**Authors:** Aleksandra Fulara, Agnieszka Hernik, Hanna Nieznańska, Wojciech Dzwolak

**Affiliations:** 1 Department of Chemistry, Biological and Chemical Research Centre, University of Warsaw, Warsaw, Poland; 2 Nencki Institute of Experimental Biology, Polish Academy of Sciences, Warsaw, Poland; University of Maryland School of Medicine, United States of America

## Abstract

Poly-L-glutamic acid (PLGA) often serves as a model in studies on amyloid fibrils and conformational transitions in proteins, and as a precursor for synthetic biomaterials. Aggregation of PLGA chains and formation of amyloid-like fibrils was shown to continue on higher levels of superstructural self-assembly coinciding with the appearance of so-called β_2_-sheet conformation manifesting in dramatic redshift of infrared amide I′ band below 1600 cm^−1^. This spectral hallmark has been attributed to network of bifurcated hydrogen bonds coupling C = O and N-D (N-H) groups of the main chains to glutamate side chains. However, other authors reported that, under essentially identical conditions, PLGA forms the conventional in terms of infrared characteristics β_1_-sheet structure (exciton-split amide I′ band with peaks at ca. 1616 and 1683 cm^−1^). Here we attempt to shed light on this discrepancy by studying the effect of increasing concentration of intentionally induced defects in PLGA on the tendency to form β_1_/β_2_-type aggregates using infrared spectroscopy. We have employed carbodiimide-mediated covalent modification of Glu side chains with n-butylamine (NBA), as well as electrostatics-driven inclusion of polylysine chains, as two different ways to trigger structural defects in PLGA. Our study depicts a clear correlation between concentration of defects in PLGA and increasing tendency to depart from the β_2_-structure toward the one less demanding in terms of chemical uniformity of side chains: β_1_-structure. The varying predisposition to form β_1_- or β_2_-type aggregates assessed by infrared absorption was compared with the degree of morphological order observed in electron microscopy images. Our results are discussed in the context of latent covalent defects in homopolypeptides (especially with side chains capable of hydrogen-bonding) that could obscure their actual propensities to adopt different conformations, and limit applications in the field of synthetic biomaterials.

## Introduction

PLGA has been a major homopolypeptide model in the field of conformational transitions in proteins since the 1950s [1]–[7]. In neutral or basic environment, repulsive culombic interactions between charged Glu side chains favor random coil conformation of the PLGA main chain. With reprotonation of side chains at low pH, a rapid coil-to-helix transition takes place [6]. However, α-helical conformation is accessible only to PLGA chains of lengths above certain critical value – acidification of short disordered oligomers of α-L-glutamic acid converts them directly into aggregated β-sheets [8]–[9], as is also the case of long-chain α-helical PLGA subjected to high temperature (e.g.[10]). The ability to adopt, depending on pH and temperature, different conformations has made PLGA an insightful model for biophysical studies on protein folding. On the other hand, the reactivity and uniformity of Glu side chains triggered interest in using PLGA and its derivatives as biodegradable drug delivery systems (e.g. [11]–[12]), components of tumor targeting gene carriers (e.g. [13]), pH-switchable multi-topology vesicles [14], and multilayer films (in complexes with polylysine) for enhancement of cell adhesion (e.g. [15]). Apart from these biomedical applications of PLGA and its derivatives, the polypeptide, along with a number of other polymerized α-amino acids, has found an important explanatory function in the context of amyloid-related disorders such as Alzheimer's or Parkinson's diseases. These maladies distinguish themselves by in vivo deposition of amyloid fibrils – abnormal linear β-sheet-rich aggregates of misfolded proteins or peptides [16]–[17]. In 2002, Fändrich and Dobson demonstrated that several homopolypetides including PLGA form amyloid-like fibrils [18]. This finding led them to propose that the capacity to form such fibrils is not restricted to a handful of disease-associated proteins but rather is a generic property of polypeptides, and is primarily driven by main-chain interactions. The authors reported infrared spectra of PLGA fibrils formed through incubation at low pD (4.08) and 65°C. In the conformation-sensitive amide I′ band vibrational region, the spectra revealed a pair of peaks at 1616 (strong) and 1683 cm^−1^ (weak) typically assigned to antiparallel intermolecular β-sheet. While these fingerprint features are commonly found in spectra of aggregated polypeptides they contrast with the infrared characteristics of similarly prepared β-aggregates described by Itoh and colleagues [19] who showed that PLGA may adopt two different antiparallel β-sheet structures: one with the spectral features similar to those described by Fändrich and Dobson (termed β_1_) and another (β_2_) with the amide I′ band position characteristically redshifted below 1600 cm^−1^. X-ray diffraction analysis pointed to different packing modes in β_1_ and β_2_ aggregates [19]. While Itoh et al. did not study the β_2_ architecture in the context of amyloid fibrils this has become subject of recent investigations carried out by our group, as well as by Keiderling′s, Kubelka's and Bouř's groups [20]–[24]. Detection of β_2_-structures through infrared absorption coincides with formation of twisted supermolecular assemblies of PLGA fibrils [21]. In all our previous investigations employing high quality linear PLGA chains [20]–[21] only β_2_-aggregates were observed, although the aggregation conditions were essentially the same or very similar to those used by Fändrich and Dobson. In our earlier studies on acid-and-temperature-induced conformational transitions in PLGA, β_1_-aggregates were detected only as metastable transient forms occurring upon co–aggregation of PLGA and PDGA (poly-D-glutamic acid) [22]. This has led us to speculate that the amyloidogenic self-assembly of poly-Glu chains unperturbed by defects, or by polydispersity of the polymer, should produce higher order structures with β_2_-like infrared characteristics. This idea also resonates with the recent study from the Keiderling's group demonstrating that synthetic (L-Glu)_10_ oligopeptide forms β_2_-fibrils [23].

The main goal of this work is to address the β_1_-vs.- β_2_ discrepancy by studying effects of increasing concentration of intentionally induced defects in PLGA on its tendency to form the two different types of aggregates. Covalent modification of Glu side chains through carbodiimide (EDC) - mediated formation of amide bonds with amines is often used for derivatization of PLGA (e.g. [25]–[26]). While the EDC-mediated chemistry is easy to control in aqueous environment in which PLGA is typically dissolved, NBA was selected on the basis of reasonable compromise between sufficient solubility in water and low violatility. In presented study, we have investigated NBA-derivatized and polylysine-doped PLGA aggregates with Fourier transform infrared (FT-IR) spectroscopy, circular dichroism (CD), scanning electron microscopy (SEM) and transmission electron microscopy (TEM).

## Materials and Methods

### Sample preparation

#### Samples

PLGA (as sodium salt, cat. No. P4761, Lot # 096K5103V, MW 15–50 kDa), PLL (as hydrobromide, cat. No. P2636, Lot # 071M5013V, MW 30–70 kDa), PDL (as hydrobromide, cat. No. P7886, Lot # SLBD8247V, MW 30–70 kDa), as well as NBA, and EDC (as N-(3–Dimethylaminopropyl)–N'-ethylcarbodiimide hydrochloride) were from Sigma, USA. D_2_O, DCl were purchased from ARMAR Chemicals, Switzerland. Branching of homopolypeptide main chains, which may occur in commercial preparations of PLGA, is undesirable, as it prevents proper folding and packing of polypeptide structures. The PLGA lot used in this work is the same as in our previous studies, and it has been extensively characterized in terms of linearity and MW [21].

### Covalent modification of PLGA

Covalent modification of PLGA side chains consisted in the following three-step procedure: first, PLGA and NBA were dissolved in a portion of D_2_O to yield molar concentrations 56 mM of PLGA's Glu side groups and 167 mM of NBA which correspond to Glu:NBA molar ratio of 1∶3. Mixed solution was then acidified with ∼1 M DCl to pD 5.3 (‘pD’ being uncorrected pH-meter readout for D_2_O-based samples). At this stage, weight concentration of PLGA was ca. 0.6%. To as-prepared sample, proper amount of fresh EDC solution in D_2_O (pD 5.3) was added, which triggered reaction leading to formation of peptide bonds between side chain Glu carboxyl groups and NBA amine groups (Supporting Information). 1 ml portions of samples containing PLGA, NBA (both at the fixed 1∶3 molar ratio), and EDC at 0.005, 0.015, 0.05, 0.15, 0.5, and 1.5 molar ratio to Glu side chains were prepared along with control PLGA sample containing neither NBA nor EDC. In the case of two samples with highest carbodiimide ratios (0.5 and 1.5), EDC solution was added to PLGA+NBA mixture gradually. Series of six samples varying in EDC concentration along with the control sample containing only PLGA were mildly agitated using magnetic stirrer for 3 h at 20°C. With the progress of chemical reaction, all samples except for the one with the highest EDC concentration (wherein extensive covalent modifications produced most hydrophobic product) remained transparent. Once the NBA/EDC-modification reaction was completed small portions of samples were used for FT-IR and CD measurements. Because NBA, EDC and products of EDC hydrolysis enable – after proper buffer subtraction – acquisition of IR spectral data in the amide I′ band region (see Supporting Information) dialysis step was omitted and FT-IR spectra of initial reaction products were taken in situ.

### Preparation of β_1_/β_2_ aggregates from covalently modified PLGA

Subsequently, samples were acidified (with diluted DCl) to pD 4.3 and subjected to 13-day-long quiescent incubation at 65°C. Under these conditions, β_1_/β_2_ aggregates precipitated from samples over time. Insoluble aggregates were centrifuged and subjected to FT-IR/TEM/SEM measurements.

### Preparation of PLGA β_1_/β_2_ aggregates containing PLL and PDL

Separately prepared fresh 1 wt. % solutions of PLGA, PLL (Poly-L-lysine), and PDL (Poly-D-lysine) in D_2_O were acidified with diluted DCl to pD 5.5. This, by partial removal of negative charges from PLGA side chains, allowed us to decelerate formation of insoluble PLGA-PLL(PDL) complexes which normally tend to form rapidly and precipitate. Hence, mildly acidified PLGA and PLL (PDL) solutions were mixed at desired ratios and were vortexed at 300 rpm and 25°C for 24 h in Eppendorf Thermomixer Comfort accessory. Subsequently, pD of all samples was further lowered to 4.1 and temperature of following quiescent incubation was increased to 60°C. After 72 h samples were subjected to FT-IR measurements.

### FT-IR Spectroscopy

For acqusition of FT-IR spectra, a CaF_2_ transmission cell and 25 mm Teflon spacer were used. Spectra were collected at 2 cm^−1^ resolution on a Nicolet NEXUS FT-IR spectrometer equipped with a liquid nitrogen-cooled MCT detector. For a single spectrum, 256 interferograms were co-added and the sample chamber was continuously purged with dry air. From each sample's spectrum corresponding buffer and water vapor spectra were subtracted. Data processing was performed with GRAMS software (ThermoNicolet, USA). All further experimental details were the same as specified earlier [20]–[21].

### Transmission Electron Microscopy

For TEM imaging of PLGA samples, 400 mesh copper grids covered with collodion (SPI Supplies, West Chester, PA, USA) and carbon were used. A 10-microliter sample (1 mg/ml) was applied to a grid for 40 s and then negatively stained for 25 s with 2 wt. % uranyl acetate (SPI Supplies). Grids were dried at room temperature and examined using JEM 1400 electron microscope from JEOL Co., Japan with high resolution digital camera (CCD MORADA, SiS-Olympus, Germany).

### Scanning Electron Microscopy

For SEM, droplets of aqueous suspensions of aggregates were deposited on silicon wafers and dried under vacuum (approx. 0.05 mbar) at room temperature. Films of fibrils were sputtered with approximately 40 nm thick layers of Au/Pd alloy before SEM images were collected on a Zeiss Leo 1530 microscope.

## Results and Discussion

The EDC-mediated modification of PLGA's Glu side chains with NBA (Figure S1) has been carried out at the fixed PLGA:NBA ratio and at varying concentrations of added EDC (Materials and Methods). PLGA does not react with NBA in the absence of EDC, and neither the amine itself nor EDC itself affect formation of β_2_-aggregates (see control experiments in Figure S2). Thus in the presence of stoichiometric excess of NBA relative to Glu side chains, the number of carboxyl groups susceptible to reaction with amine is effectively controlled by the concentration of EDC. As the reaction leads to more hydrophobic and no longer ionizable side chains, it affects properties of PLGA even before polypeptide is converted into β-aggregates.

**Figure 1:**
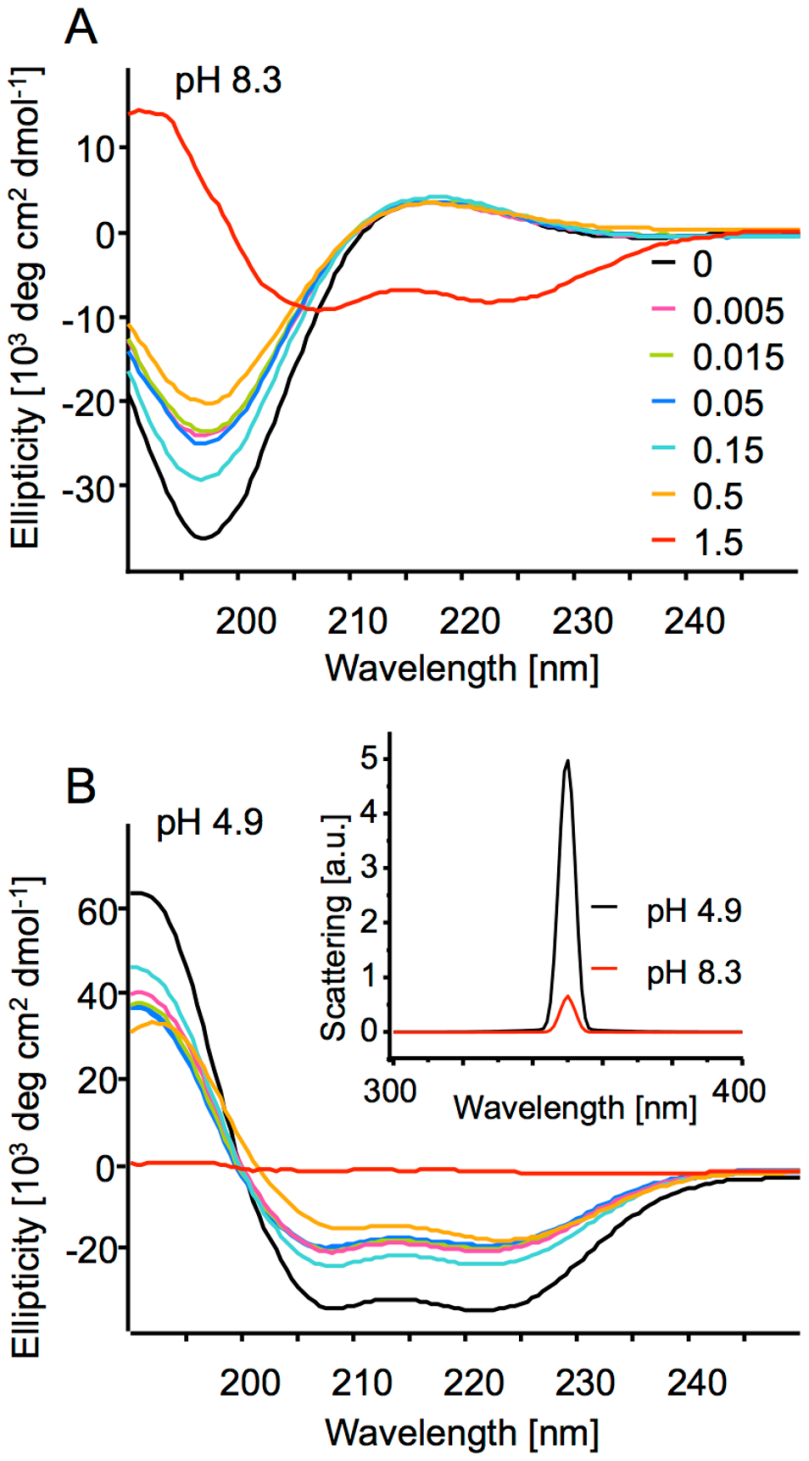
Spectral characteristics of NBA/EDC-modified PLGA samples. Far-UV CD spectra of PLGA samples modified with NBA (at fixed1∶3 Glu side chain: NBA molar ratio) in the presence of varying concentrations of EDC (expressed as molar ratio of EDC:Glu side chains: 0, 0.005, 0.015, 0.05, 0.15, 0.5, and 1.5) after alkalization to pH 8.3 (A) and subsequent acidification to pH 4.9 (B). Changes in light scattering intensity (at 350 nm) of NBA/EDC-modified PLGA formed at 1.5 EDC:Glu molar ratio caused by pH-adjustment are shown in the inset in panel (B).

Figure 1 shows far-UV CD spectra of fresh PLGA samples progressively modified with NBA. The spectra were collected after initial alkalization of samples to pH 8.3 (A) and also after subsequent acidification to pH 4.9. (B). At any pH above 6, unmodified PLGA acquires random coil conformation (e.g. [27]), which is also the case to NBA-modified samples at EDC:Glu ratios 0.5 and lower – reflected by the characteristic negative at 197 nm and slightly positive at 217 nm spectral curvature visible in Figure 1A. However, the most profoundly modified sample (1.5 EDC:Glu ratio) shows double 208/222 nm minima which are distinctive for α-helical conformation – normally not accessible to PLGA at this high pH. This is a likely consequence of the fact that the extensive modification of Glu side chains causes simultaneous desensitization of PLGA to pH – the limited ionization of the polypeptide no longer prevents folding. CD spectra were again measured after pH of each sample was lowered to 4.9 (Figure 1B). All samples except for the most modified one (for which the spectrum is essentially flat) reveal well-defined α-helical signals. Because the acidification led to partial precipitation of the ‘1.5 EDC’ sample – reflected by the increase of light scattering at 350 nm (inset in Figure 1B), the lack of interpretable CD signals in the corresponding spectrum should be attributed only to poor optical quality of the sample.

**Figure 2:**
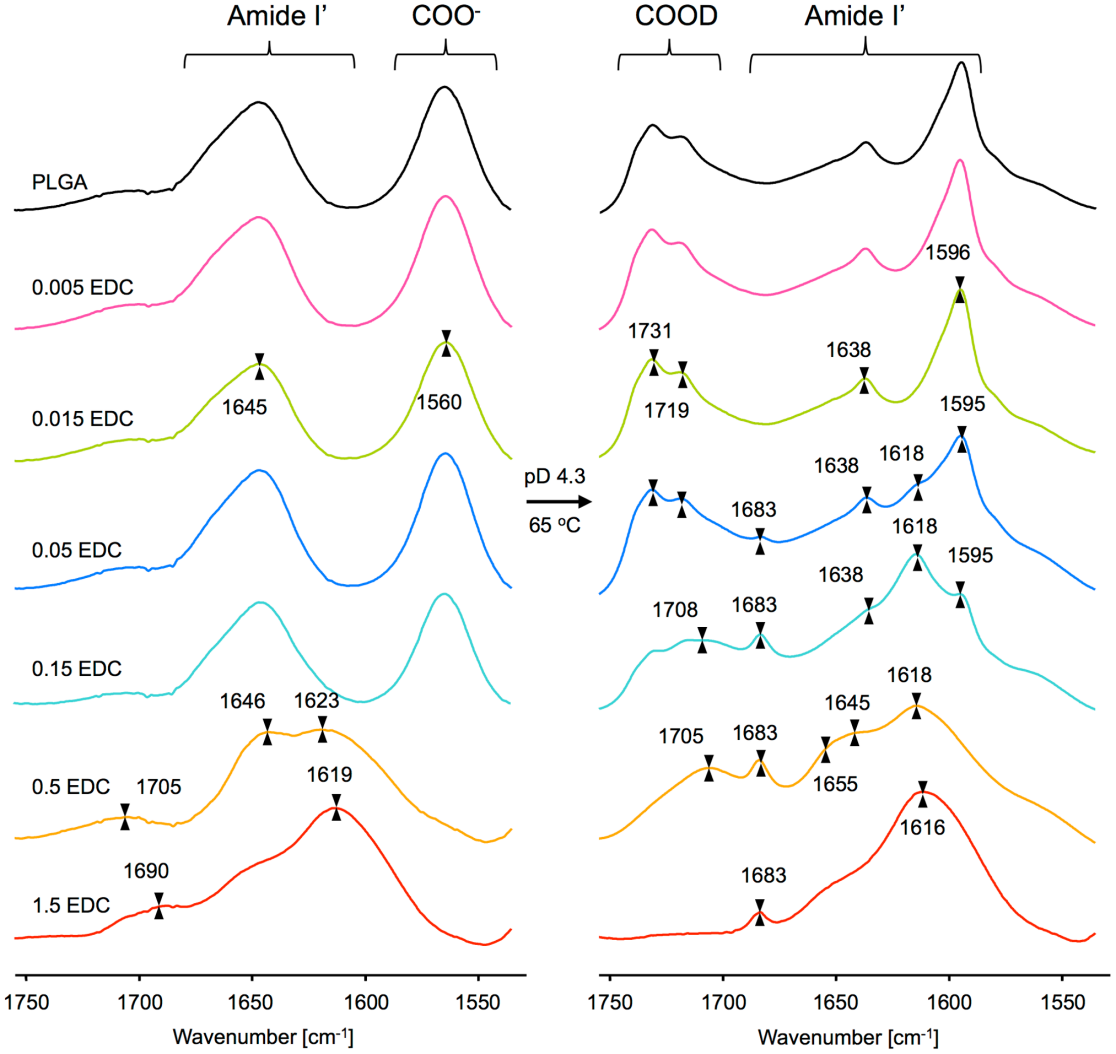
Infared spectra of PLGA samples right after covalent modifications with NBA at indicated EDC:Glu molar ratios (left panel), and after subsequent acidification to pD 4.3 and 13-day-long incubation at 65°C (right panel). The data corresponds to several parallel experiments.

Preliminary characterization of modified PLGA samples captures certain differences relative to unmodified PLGA which are rather intuitive. The thus obtained and characterized samples were used as precursors for preparation of β-aggregates. This was carried out by further lowering pD to 4.3 followed by prolonged incubation at 65°C. Importantly, IR spectra of NBA, EDC, and products of EDC hydrolysis either do not overlap the amide I′ band region, or are easy to subtract from polypeptide spectra (Figure S3). This coupled to D_2_O being used as the reaction allowed us to probe the modification process in situ even before the final acidification and incubation step (when β_1_/β_2_ aggregates precipitate and become very easy to separate from the solution). FT-IR spectra of increasingly modified PLGA samples before and after the aggregation step are juxtaposed in Figure 2. In agreement with the CD data, at the close-to-neutral pD of the reaction, slightly modified PLGA samples retain random coil conformation with the corresponding amide I′ band at 1645 cm^−1^ and prominent 1560 cm^−1^ band assigned to antisymmetric stretching vibrations of Glu –COO– groups [28]. Only at 0.5 EDC:Glu ratio the latter band decreases in intensity (as the ionizable carboxyl groups are replaced with peptide bonds) while the amide I′ band splits into two components at 1646 and 1623 cm^−1^. At the highest EDC concentration, the amide I′ band becomes further shifted down to 1619 cm^−1^ with an overlapping broad component at approximately 1650 cm^−1^ and another tiny and poorly resolved peak at 1690 cm^−1^. The simultaneous appearance of strong spectral component below 1620 cm^−1^ and weaker one above 1680 cm^−1^ is routinely assigned to excitonic splitting taking place in an intramolecular antiparallel β-sheets (type β_1_) – B(π,0) and B(0,π) modes, respectively [29]. Certainly, absorption from newly-created amide bonds in NBA-modified PLGA side chains will overlap these main-chain signals. In this respect, it should be mentioned that the amide I′ band of N-methylacetamide dissolved in D_2_O is centered just above 1621 cm^−1^ [33]. However, for the case studied in this work, reference data on isolated secondary amides is of limited use as the actual spectral contribution from the side chains may strongly depend on their local self-association behavior (and surroundings within the aggregate).

The key result of this study is presented in Figure 2B which shows FT-IR spectra of modified PLGA samples after acidification and prolonged incubation at 65°C. The most singular aspect of the IR characteristics of β_2_-structure of PLGA is the pair of amide I′ band component peaks: a small one at ca. 1638 cm^−1^ and the large one shifted to 1596 cm^−1^. At this low pD Glu side chains undergo deuteration: -COOD band appears above 1700 cm^−1^ at the expense of the 1560 cm^-1^ band corresponding to –COO– stretches. Only in β_2_-type aggregates of PLGA the –COOD band is characteristically split into two peaks at approximately 1731 and 1719 cm^−1^ [20]–[21]. The extreme redshift of the amide I′ band in β_2_–PLGA was attributed to reduced strength of amide C = O bonds upon formation of unusual three-center hydrogen bonds involving bifurcated main chain carbonyl acceptors and main chain ND (NH), as well as side chain –COOD (–COOH) donors [20]. According to the data in Figure 2B these spectral features are strictly maintained for aggregates of partly modified PLGA chains at the two lowest EDC concentrations (0.005 and 0.015 EDC:Glu ratios). However, a more extensive modification of PLGA at the even higher EDC concentration (EDC:Glu ratio  =  0.05), results in the emergence of peaks at 1618 and 1683 cm^−1^ and broadening of the –COOD band. More remarkable spectral changes are seen for aggregates of PLGA chains modified in the presence of ‘0.15 EDC’. The pair of 1618/1683 peaks corresponding to β_1_-type dominates the amide I′ region while the –COOD bandshape is deformed and reduced in intensity. Interestingly, this degree of modification of side chains does not affect so dramatically PLGA before the aggregation (spectra in panels A and B) implying a higher sensitivity of β_2_-architecture to such defects. The β_2_-components do not appear in the spectra of aggregated chains modified at the two highest EDC concentrations (‘0.5’ and ‘1.5’). Several factors are likely to contribute to the strong broadening of the amide I′ band including local conformational disorder, polydispersion of β_1_-aggregates, overlapping absorption of side chains′ peptide bonds and different degree of solvent-exposure of surface-, and buried main chain amide bonds. The most thoroughly modified PLGA chains assemble into neat β_1_-structure reflected by the pair of 1616 and 1683 cm^−1^ peaks and the corresponding spectra appear to be least affected by the acidification/aggregation procedure of all examined PLGA samples.

**Figure 3:**
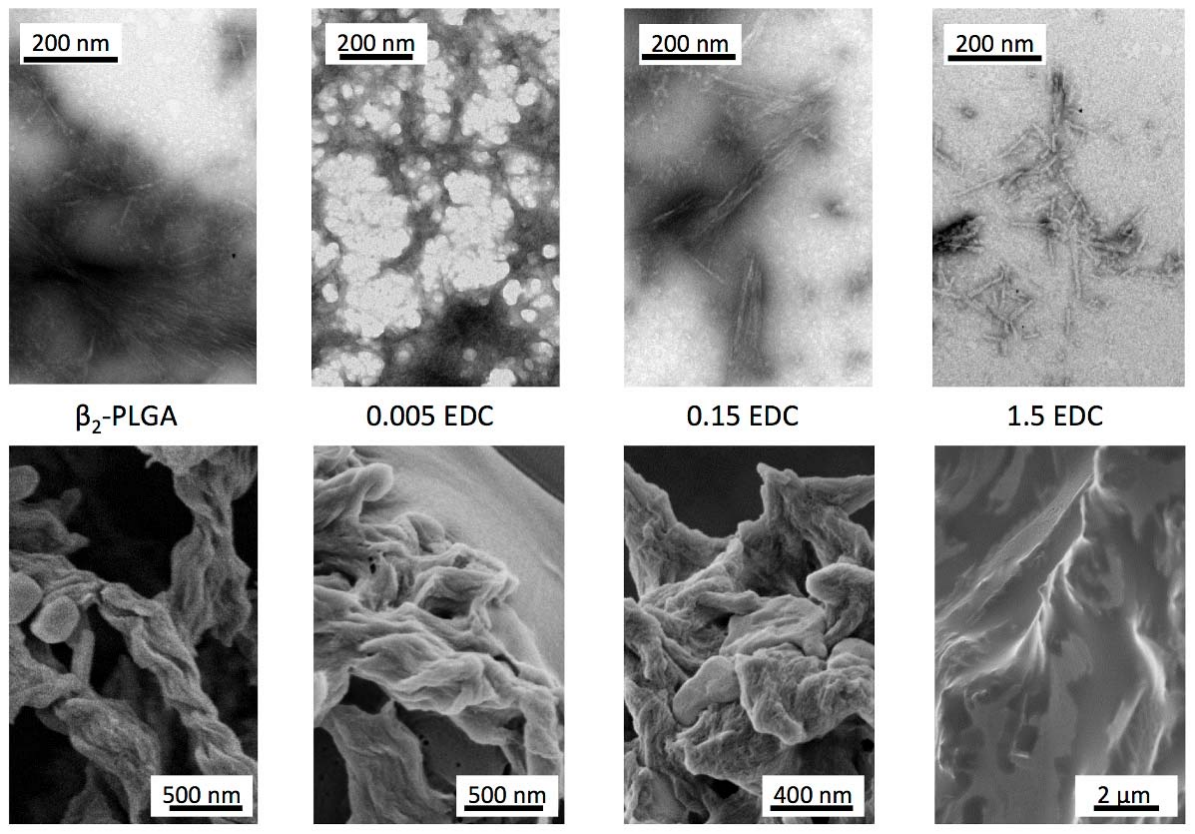
TEM (top row) and SEM (bottom row) images of amyloid fibrils formed by unmodified PLGA (β_2_) and selected NBA/EDC-modified PLGA samples.

Because random modifications of homopolypeptide side chains could, in principle, lead to complete disorder on all levels of the structural organization of aggregates, we have analyzed morphologies of β_1_/β_2_ samples. TEM (after negative staining - upper row) and SEM (bottom row) images of selected aggregates are shown in Figure 3. Clearly, the increasing degree of modification of side chains leads to rapid decrease in number of long helically-twisted aggregates, which are prominent only in SEM image of unmodified β_2_-PLGA. Even the mildest modification in the presence of ‘0.005’ EDC appears to limit the long-range order of these assemblies. Interestingly, on the lower level of assembly corresponding to single PLGA fibrils (visible in TEM), the structural order appears to be preserved also in the case of ‘1.5’-EDC-modified aggregates.

**Figure 4:**
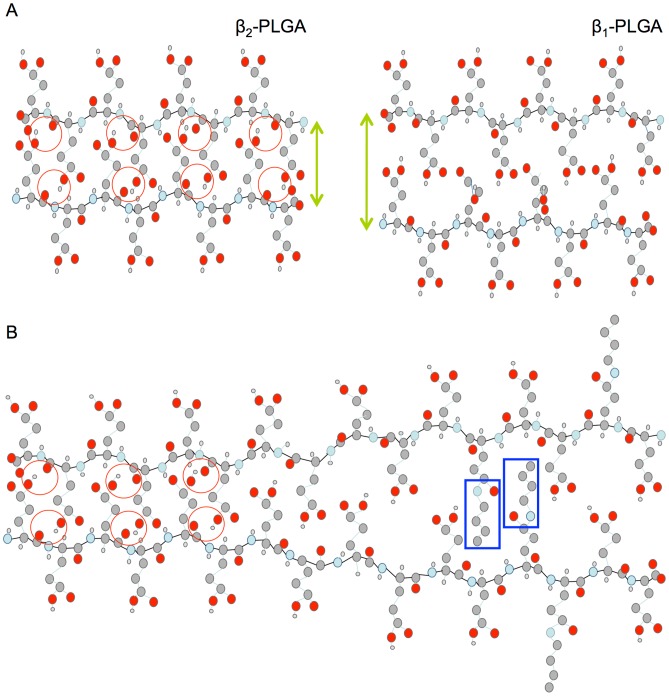
Cross-section view at a model of different inter-sheet distances and packing modes of Glu side chains in the β_1_/β_2_-type structural variants of PLGA aggregates with the antiparallel arrangement of strands (A). Red circles mark sites of three-center hydrogen bonds with bifurcated carbonyl acceptors. Random covalent modification of Glu side chains (within frames) cause local structural defects and result in less-densely-packed β_1_ fibrils (B).

**Figure 5:**
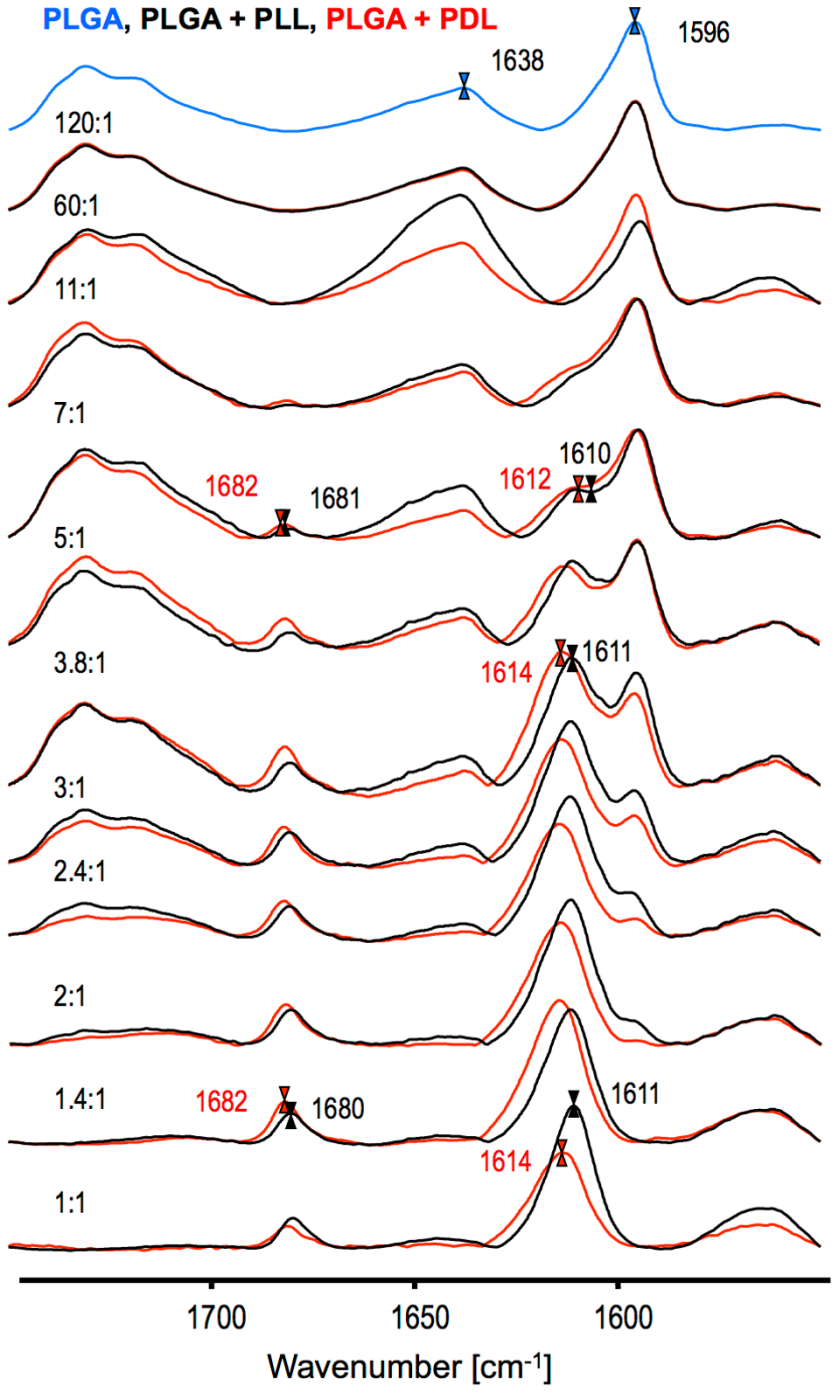
Infrared spectra of PLGA aggregates doped with PLL (black lines), or PDL (red lines) at the indicated Glu:Lys side chain molar ratios. Aggregates were formed by incubation (72h/60°C) of acidified mixtures of PLGA and PLL (PDL). Blue spectrum corresponds to β_2_-fibrils formed in the absence of polylysine.

The spectral data presented in Figure 2B strongly supports the idea that β_2_-fibrils are the proper end product of defect-free amyloidogenic self-assembly of PLGA chains [20]–[22]. Formation of fibrils with β_2_-type infrared features requires the occurrence of additional stabilizing interactions: bifurcated hydrogen bonds involving Glu carboxyl groups and main chain carbonyl groups. This may explain the earlier observations that in mixtures of PLGA and PDGA chains, β_2_-fibrils appear to be more thermodynamically stable than β_1_-aggregates [22]. Certainly, the dense packing and strict steric requirements for three-center hydrogen bonds narrowly define the structure of β_2_-fibrils. On the other hand, aggregates with the β_1_-like spectral features are more likely to constitute a broad ensemble of agglomerates unable to establish such bonding patterns for one reason (kinetic traps caused by co-aggregation of PLGA and PDGA chains [22]) or another (e.g. random covalent modifications of side chains used in this study). The spectral and SEM data shown in Figures 2 and 3 reflect high sensitivity of β_2_-fibrils to structural defects. Even substoichiometric modifications of side chains result in barriers against formation of three-centered hydrogen bonds not only at the modification site, but most likely (through local mechanical strain and spatial separation of sheets – as explained in Figure 4) also in direct vicinity. It should be stressed that there are many possible perturbation scenarios for the self-assembly of β_2_-fibrils. Here, we have employed covalent modification of side-chains with an arbitrarily selected amine. Arguably different amines could prove more effective in inducing structural defects in β_2_-fibrils, as also could be the case of sporadic branching of main chains [30]. Non-covalent binding and inclusion of molecules sterically incompatible with this type of PLGA self-assembly could prove yet another way of disrupting the β_2_-architecture. We have tested this hypothesis using PLL and PDL as model disruptors, since polylysine is known to form stable β-pleated electrostatic complex with PLGA [31]–[32]. Figure 5 shows FT-IR spectra of PLGA doped with either PLL or PDL at different molar ratios (expressed as Glu:Lys side chains). The β_1_-type spectral features set in at approximately 11-fold excess of PLGA and begin to dominate over β_2_ when this excess is lowered to 3.8∶1 – clearly, addition of polylysine prevents formation of PLGA β_2_-fibrils at substoichiometric levels. The minor spectral shifts of PLGA-PLL relative to PLGA-PDL complexes are due to locally different β-sheet geometries and have been described before [32].

Aggregation and amyloidogenic self-assembly of linear and undamaged PLGA chains appears to lead exclusively to β_2_-type fibrils. The process is very sensitive to structural perturbations which may consist either in covalent modifications of side chains or entrapment of foreign molecules leading to aggregates with the regular amide I/I′ band traits associated with intermolecular beta sheets. One could speculate that the outcome of at least some of published studies on PLGA aggregates revealing only β_1_-type spectra (formed in the apparent absence of such purposefully induced perturbations) might have been influenced by contaminations or unsuccessful synthesis (ineffective removal of protecting groups from side chains or branching of main chains). Interestingly, out of several homopolypeptides with side chains capable of hydrogen bonding and therefore potentially favoring formation of β_2_-like aggregates (along with poly-L-lysine, poly-L-threonine, poly-L-aspartic acid) such forms were only observed for PLGA. To what extent this was influenced by chemical imperfection of synthetic homopolypeptides remains unclear. This also underlies urgency to revisit several decades-old works describing limits to structural transitions and superstructural self-assembly of homopolypetides.

## Conclusions

Unperturbed aggregation of defect-free PLGA chains ultimately leads to superstructures of amyloid-like fibrils with unusual infrared characteristics in the amide I′ band region, so-called β_2_-fibrils. With increasing number of structural defects introduced either by covalent modification of Glu side chains or through non-covalent co-binding of foreign peptides PLGA aggregates progressively depart from the β_2_-type towards less-ordered forms with β_1_-type spectral features that are commonly associated with intermolecular antiparallel β-sheet. Our study highlights the importance of thorough physicochemical characterization of polymerized-α-L-amino acids used in basic and applied research.

## Supporting Information

Figure S1 **The procedure of introducing defects in PLGA by covalent modifications of Glu side chains and primary amines mediated by EDC.** Solutions of PLGA and NBA in D_2_O were mixed (at 1∶3 Glu side chain: NBA molar ratio), followed by pH* adjustment to 5.3. Subsequently, the reaction was initiated by adding desired amounts of EDC. The mixture was stirred for 3 h at 20°C.
(PDF)

Figure S2 FT-IR spectra of PLGA incubated with (A) NBA (at 1∶3 Glu-side-chain:NBA molar ratio); (B) EDC (at 1∶1.5 Gluside-chain:EDC molar ratio); (C) NBA and EDC (at 1∶3∶1.5 Glu-side-chain:NBA:EDC molar ratio), in D_2_O at pH* 4.3 65°C for 13 days. Blue rectangle marks amide I/I′ band region.
(PDF)

Figure S3 FT-IR spectra of NBA, fresh EDC, and hydrolyzed EDC, all dissolved in D_2_O at pH* 5.3 (solvent-subtracted spectra). Hydrolysis of EDC was carried out by mixing 0.1 M EDC with equimolar amount of DCl followed by 3 h incubation at 20°C, pH* was re-adjusted to 5.3 prior to FT-IR measurements. Blue rectangle marks amide I/I′ band region.
(PDF)
