# Supplementary material for: Covalent Defects Restrict Supramolecular Self-Assembly of Homopolypeptides: Case Study of β2-Fibrils of Poly-L-Glutamic Acid
Source: PLoS One. 2014 Aug 21;9(8):e105660. doi: 10.1371/journal.pone.0105660 (PMC4140804; doi:10.1371/journal.pone.0105660)
Supplement: Figure S1 — The procedure of introducing defects in PLGA by covalent modifications of Glu side chains and primary amines mediated by EDC. Solutions of PLGA and NBA in D2O were mixed (at 1∶3 Glu side chain: NBA molar ratio), followed by pH* adjustment to 5.3. Subsequently, the reaction was initiated by adding desired amounts of EDC. The mixture was stirred for 3 h at 20°C. (PDF) [file pone.0105660.s001.pdf]

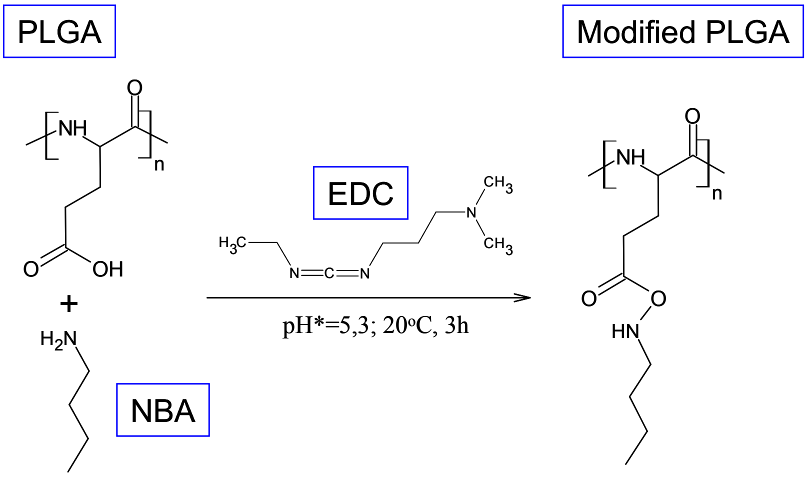

Figure S1.
The procedure of inducing defects in PLGA by covalent modifications of Glu side chains with EDC and primary amines explained on NBA example. Solutions of PLGA and NBA in D_2O were mixed (at 1:3 Glu side chain : NBA molar ratio), followed by pH* adjustment to 5.3. Subsequently, the reaction was initiated by adding desired amounts of EDC and stirring at 20 °C for 3 h.
