# Supplementary material for: Covalent Defects Restrict Supramolecular Self-Assembly of Homopolypeptides: Case Study of β2-Fibrils of Poly-L-Glutamic Acid
Source: PLoS One. 2014 Aug 21;9(8):e105660. doi: 10.1371/journal.pone.0105660 (PMC4140804; doi:10.1371/journal.pone.0105660)
Supplement: Figure S2 — FT-IR spectra of PLGA incubated with (A) NBA (at 1∶3 Glu-side-chain:NBA molar ratio); (B) EDC (at 1∶1.5 Gluside-chain:EDC molar ratio); (C) NBA and EDC (at 1∶3∶1.5 Glu-side-chain:NBA:EDC molar ratio), in D2O at pH* 4.3 65°C for 13 days. Blue rectangle marks amide I/I′ band region. (PDF) [file pone.0105660.s002.pdf]

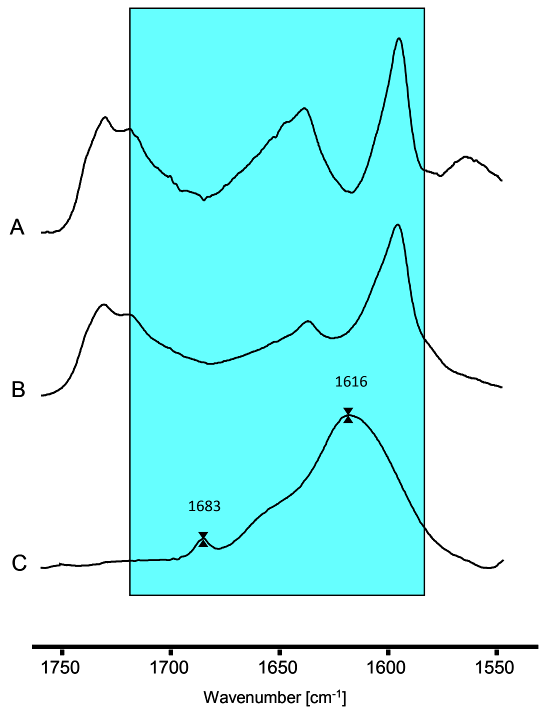

Figure S2.
FT-IR spectra of: (A) PLGA incubated with NBA (at 1:3 Glu-side-chain:NBA molar ratio) in D_2O at pH* 4.3 / 65˚C; (B) PLGA incubated with EDC (at 1:1.5 Glu-side-chain:EDC molar ratio) in D_2O at pH* 4.3 / 65˚C; (C) PLGA incubated with NBA and EDC (at 1:3:1.5 Glu-side-chain:NBA:EDC molar ratio) in D_2O at pH* 4.3 / 65˚C. Blue rectangle marks amide I/I' band region.
