# Supplementary material for: Covalent Defects Restrict Supramolecular Self-Assembly of Homopolypeptides: Case Study of β2-Fibrils of Poly-L-Glutamic Acid
Source: PLoS One. 2014 Aug 21;9(8):e105660. doi: 10.1371/journal.pone.0105660 (PMC4140804; doi:10.1371/journal.pone.0105660)
Supplement: Figure S3 — FT-IR spectra of NBA, fresh EDC, and hydrolyzed EDC, all dissolved in D2O at pH* 5.3 (solvent-subtracted spectra). Hydrolysis of EDC was carried out by mixing 0.1 M EDC with equimolar amount of DCl followed by 3 h incubation at 20°C, pH* was re-adjusted to 5.3 prior to FT-IR measurements. Blue rectangle marks amide I/I′ band region. (PDF) [file pone.0105660.s003.pdf]

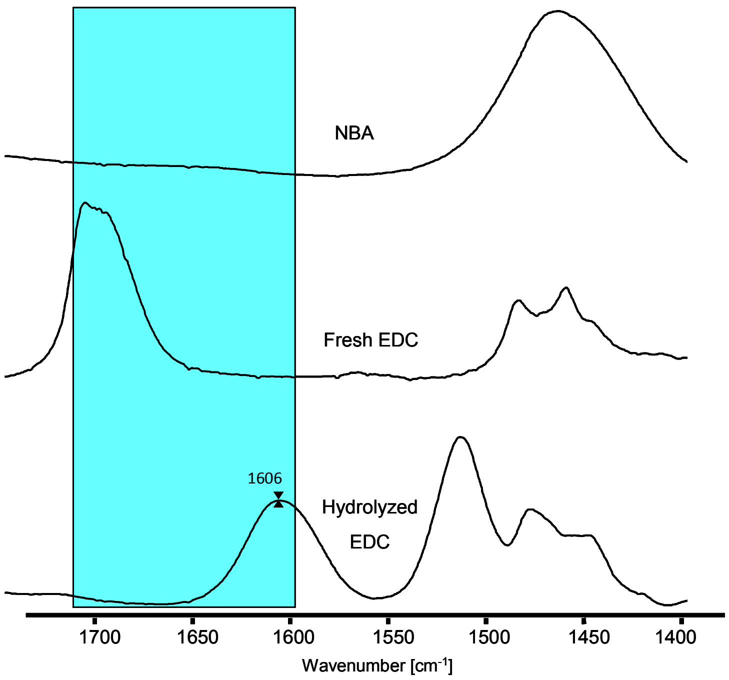

Figure S3.
FT-IR spectra of NBA, fresh EDC, and hydrolyzed EDC, all dissolved in D_2O at pH* 5.3 (solvent-subtracted spectra). Hydrolysis of EDC was carried out by mixing 0.1 M EDC with equimolar amount of DCl followed by 3h incubation at 20˚C, pH* was re-adjusted to 5.3 prior to FT-IR measurements. Blue rectangle marks amide I/I' band region.
